# Efficacy of neoadjuvant pembrolizumab combined with paclitaxel and cisplatin in locally advanced oropharyngeal and hypopharyngeal squamous cell carcinoma: a retrospective study

**DOI:** 10.3389/fimmu.2025.1690935

**Published:** 2025-11-21

**Authors:** Shupin Tang, Gongbiao Lin, Xiaobo Wu, Zhihong Chen

**Affiliations:** 1 Department of Otolaryngology Head and Neck Surgery, the First Affiliated Hospital, Fujian Medical University, Fuzhou, China; 2 Department of Otolaryngology Head and Neck Surgery, National Regional Medical Center, Binhai Campus of the First Affiliated Hospital, Fujian Medical University, Fuzhou, China; 3 Fujian Institute of Otorhinolaryngology, the First Affiliated Hospital, Fujian Medical University, Fuzhou, China; 4 Fujian Clinical Research Center for Difficult Otorhinolaryngologic Diseases, the First Affiliated Hospital, Fujian Medical University, Fuzhou, China

**Keywords:** locally advanced oropharyngeal and hypopharyngeal cancer, immune checkpoint inhibitors, neoadjuvant therapy, chemoimmunotherapy, transoral surgery

## Abstract

**Objective:**

This retrospective study aims to evaluate the efficacy and safety of pembrolizumab combined with paclitaxel and cisplatin (ICI+TP) in the treatment of locally advanced oropharyngeal and hypopharyngeal squamous cell carcinoma (OPHSCC).

**Methods:**

Patients with locally advanced OPHSCC who received ICI+TP, cetuximab with paclitaxel-cisplatin (CET+TP), or paclitaxel-cisplatin alone (TP) were reviewed. Radiographic and pathological response rates, transoral surgery rates, and survival outcomes were assessed.

**Results:**

A total of 83 patients were enrolled in this study, with 23 subjected to ICI+TP, 31 to CET+TP, and 29 to TP. Compared with TP, ICI+TP yielded superior clinical outcomes: a higher objective response rate (ORR) (91.3% vs. 55.2%, *p*<0.05), greater transoral surgery feasibility (91.3% vs. 34.5%, *p*<0.05), lower tracheostomy incidence (26.1% vs. 79.3%, *p*<0.05). Among hypopharyngeal carcinoma patients, ICI+TP achieved an ORR of 100%, outperforming both CET+TP (62.5%) and TP (51.9%). The transoral surgery rate reached 89.5% (vs. 29.6% with TP, *p*<0.05) while tracheostomy requirements were reduced (21.1% vs. 85.2%, *p*<0.05). Notably, ICI+TP produced significantly higher primary tumor pathological complete response rates than CET+TP (57.9% vs. 20.8%, *p*<0.05). Median follow-up was 10 months for ICI+TP, 13 months for CET+TP, and 24 months for TP. Neither progression-free nor overall survival showed significant improvement among the three groups.

**Conclusion:**

In locally advanced OPHSCC, neoadjuvant pembrolizumab combined with paclitaxel and cisplatin showed a higher ORR and increased transoral surgery rates while preserving laryngeal function, with no increase in severe treatment-related adverse events, demonstrating favorable efficacy and safety profiles.

## Introduction

Head and neck cancer ranks as the sixth most common cancer and the seventh leading cause of cancer-related death among Chinese males (1). Head and neck squamous cell carcinoma (HNSCC) accounts for approximately 90% of these cases (2). Hypopharyngeal squamous cell carcinoma (HPSCC) makes up less than 5% of all head and neck malignancies. Notably, oropharyngeal squamous cell carcinoma (OPSCC) has seen a rising incidence over the past two decades, particularly HPV-positive cases among non-smokers and non-drinkers (3–5). Early-stage hypopharyngeal and oropharyngeal carcinomas often present with non-specific symptoms. This frequently leads to diagnosis at an advanced stage, accompanied by extensive lymph node involvement. Despite the use of multimodal therapies combining surgery, radiation, and chemotherapy, locally advanced HNSCC still carries substantial risks of laryngeal dysfunction, disease recurrence, and distant metastasis, with five-year survival rates remaining 40-50% (6, 7).

Recent advances in immunotherapy have transformed HNSCC management. Tumor cells evade immune surveillance by activating multiple immunosuppressive checkpoint pathways, among which programmed cell death protein 1 (PD-1) and its ligands act as key regulators. Within the tumor microenvironment (TME), tumor cells often exhibit high expression of programmed cell death 1 ligand 1 (PD-L1), driven by mechanisms such as gene amplification and induction by inflammatory cytokines. These PD-L1 molecules engage with PD-1 on tumor-infiltrating T cells, transmitting sustained inhibitory signals. As a result, T cell proliferation is impaired, secretion of cytotoxic factors including interferon-γ (IFN-γ) and tumor necrosis factor-α (TNF-α) is reduced, and T cells gradually enter an exhausted state, ultimately losing their tumoricidal capacity. Additionally, PD-L1 expressed by myeloid-derived suppressor cells (MDSCs) can also bind PD-1 on T lymphocytes. This impairs T cell-mediated antitumor immunity and suppressing natural killer (NK) cell activity, thereby promoting tumor progression (8). Immune checkpoint inhibitors enhance T-cell proliferation and antitumor responses by blocking PD-1/PD-L1 interactions, demonstrating considerable clinical potential (9). The KEYNOTE-048 trial established pembrolizumab combined with platinum-based chemotherapy as first-line therapy for recurrent/metastatic HNSCC, on the basis of significant survival benefits (10, 11). This success has spurred investigation into combining PD-1 inhibitors with neoadjuvant chemotherapy for locally advanced disease, aiming to improve surgical outcomes, reduce metastasis risk, and preserve organ function. Preliminary findings from phase II trials—including those evaluating toripalimab in combination with paclitaxel/cisplatin and camrelizumab with nab-paclitaxel/cisplatin regimens—demonstrate encouraging response rates and manageable toxicity profiles (2, 12). Retrospective analyses further suggest neoadjuvant chemoimmunotherapy may prolong progression-free survival (PFS) and overall survival (OS) in OPSCC (13), albeit long-term outcome data remain limited.

This study aims to evaluate the efficacy and safety of pembrolizumab combined with neoadjuvant chemotherapy in patients with resectable locally advanced oropharyngeal and hypopharyngeal squamous cell carcinoma (OPHSCC), by comparing it with cetuximab plus neoadjuvant chemotherapy and neoadjuvant chemotherapy alone.

## Methods

### Patients and data collection

**Figure 1:**
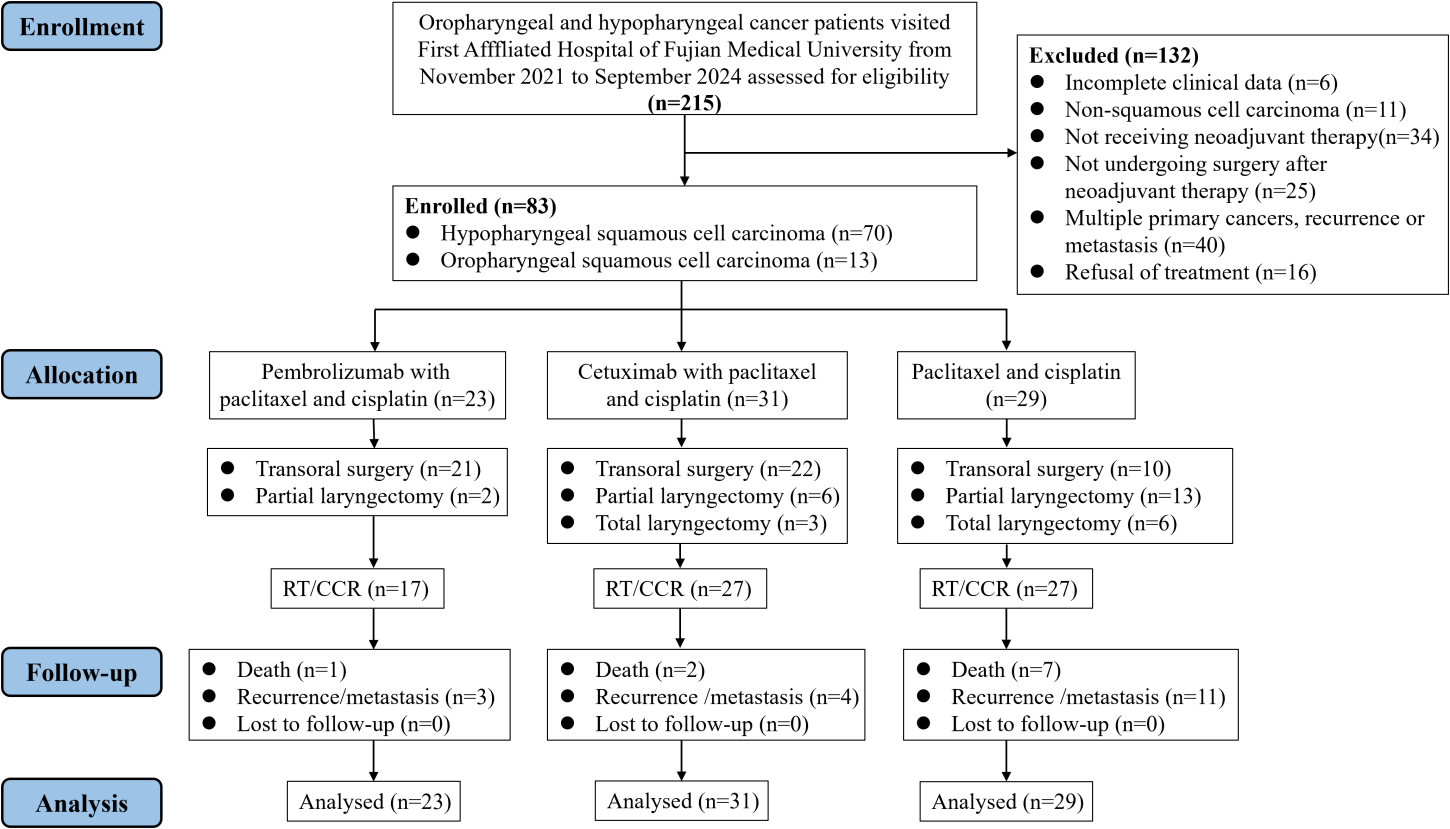
CONSORT-style flow diagram for inclusion of patients with OPHSCC. Partial laryngectomy: partial laryngectomy with partial hypopharyngectomy; Total laryngectomy: total laryngectomy with partial hypopharyngectomy. RT, radiotherapy; CCR, concurrent chemoradiotherapy.

A total of 215 patients diagnosed with oropharyngeal and hypopharyngeal cancer were identified and screened from the hospital database over the period between November 2021 and September 2024. Of these, 83 patients were ultimately included in the final analysis. Inclusion criteria required patients to: (1) be aged ≥18 years; (2) have treatment-naive hypopharyngeal or oropharyngeal carcinoma; (3) have histologically confirmed squamous cell carcinoma; (4) exhibit stage III or IV disease according to the 8th edition of the AJCC criteria; (5) complete at least one cycle of pembrolizumab plus neoadjuvant chemotherapy, cetuximab plus neoadjuvant chemotherapy, or neoadjuvant chemotherapy alone; and (6) undergo post-neoadjuvant surgery. Exclusion criteria comprised: (1) incomplete clinical data; (2) non-squamous cell carcinoma; (3) not receiving neoadjuvant therapy; (4) not undergoing surgery after neoadjuvant therapy; (5) recurrent or metastatic disease; (6) synchronous primary tumors at other sites; (7) drug allergies relevant to the study; (8) active pneumonia or autoimmune disease; (9) general anesthesia contraindications; (10) refusal of treatment. The detailed process is shown in **Figure 1**.

All participants underwent pretreatment evaluation including blood tests, laryngoscopy, and contrast-enhanced computed tomography (CT)/magnetic resonance imaging (MRI) of the head and neck. Baseline positron emission tomography-computed tomography (PET-CT) excluded metastatic disease, while esophagogastroduodenoscopy screened hypopharyngeal cancer patients for synchronous esophageal/gastrointestinal malignancies.

The study protocol was reviewed and approved by the Ethics Committee of First Affiliated Hospital of Fujian Medical University (Certificate NO.: (2015)084-2). All patients provided written informed consent prior to treatment initiation.

### Treatment regimens

Patients were stratified into three treatment cohorts: pembrolizumab with paclitaxel-cisplatin (ICI+TP), cetuximab with paclitaxel-cisplatin (CET+TP), or paclitaxel-cisplatin alone (TP). The ICI+TP regimen administered pembrolizumab (200 mg, day 1) followed by paclitaxel (135 mg/m²) and cisplatin (35 mg/m², day 2), with additional cisplatin (35 mg/m², days 3-4). The CET+TP protocol delivered cetuximab (400 mg/m²) alongside paclitaxel (135 mg/m²) and cisplatin (35 mg/m², day 1), supplemented by cisplatin (35 mg/m², days 2-3). The TP group received paclitaxel (135 mg/m²) and cisplatin (35 mg/m², day 1) plus cisplatin (35 mg/m², days 2-3). All regimens involved 3-week cycles, with at least one cycle completed before evaluation. Patients who failed to achieve complete response (CR) or partial response (PR) after one cycle, declined immediate surgery, or tolerated treatment-related adverse events proceeded to second or third neoadjuvant cycles. Following neoadjuvant therapy, surgical resection of primary tumors was performed via transoral radiofrequency coblation resection, partial or total laryngectomy with partial hypopharyngectomy, and concurrent neck lymph node dissection.

### Efficacy assessments

Clinical radiographic response to neoadjuvant treatment was assessed using Response Evaluation Criteria in Solid Tumors (RECIST 1.1), categorizing outcomes as CR, PR, progressive disease (PD), or stable disease (SD) (14). The objective response rate (ORR) comprised patients achieving CR or PR (15, 16). Pathological assessment examined resected primary lesions and lymph node specimens postoperatively. Pathological complete response (pCR) was defined as 0% residual viable tumor cells in the primary tumor and sampled lymph nodes. Major pathological response (MPR) was defined as ≤10% residual viable tumor cells in the primary tumor and sampled lymph nodes. Incomplete pathological response (IPR) was defined as the presence of >10% viable tumor cells in the primary tumor and sampled lymph nodes (17).

### Follow-up

Patients underwent regular follow-up assessments either in the outpatient department or via telephone. These evaluations included laryngoscopy, contrast-enhanced MRI of the head and neck region, and CT scans of the thorax and abdomen. OS was calculated from the start of neoadjuvant therapy until death from any cause. PFS was measured from treatment initiation until either radiographic disease progression or death (18).

### Statistical analyses

Statistical analyses were conducted using SPSS 26.0 (IBM). Continuous variables were analyzed with Student’s t-test or the Mann-Whitney U test, while categorical variables were compared using the Chi-square test or Fisher’s exact test. Univariate and multivariate logistic regression models were employed to identify independent predictors of pCR. The Kaplan-Meier method was used to generate survival curves and estimate OS and PFS rates, with differences between curves assessed using the log-rank test. Univariate and multivariate Cox proportional hazards regression models were utilized to evaluate independent prognostic factors for survival. Variables with a *p*-value < 0.05 in the univariate analysis were included in the subsequent multivariate model. The Bonferroni method was applied for multiple testing correction. Graphs were generated using GraphPad Prism 8.0 and Adobe Illustrator CS6 software.

## Results

### Clinical characteristics of the study cohort

This study included 83 patients (74 male, 9 female) with a mean age of 60.7 years (range: 37-78). The primary tumor originated in the hypopharynx (84.3%) or oropharynx (15.7%). All cases presented with locally advanced disease, including stage IV tumors (68.7%), while preoperative imaging detected lymph node metastasis in 89.2% of patients. Neoadjuvant regimens consisted of ICI+TP (n=23), CET+TP (n=31), or TP alone (n=29). Surgical interventions included transoral resection (n=53), partial laryngectomy with partial hypopharyngectomy (n=21), or total laryngectomy with partial hypopharyngectomy (n=9). Postoperative radiotherapy was delivered to 85.5% of patients (**Table 1**).

**Table 1:** Baseline characteristics of the study cohort.

Characteristic	ICI+TP (n=23)	CET+TP (n=31)	TP (n=29)	Total (n=83)	*P* value
Age, years					0.467
<65	12 (52.2)	21 (67.7)	19 (65.5)	52 (62.7)	
≥65	11 (47.8)	10 (32.3)	10 (34.5)	31 (37.3)	
Gender (%)					0.412
Male	22 (95.7)	26 (83.9)	26 (89.7)	74 (89.2)	
Female	1 (4.3)	5 (16.1)	3 (10.3)	9 (10.8)	
Primary site (%)					0.241
Hypopharynx	19 (82.6)	24 (77.4)	27 (93.1)	70 (84.3)	
Oropharynx	4 (17.4)	7 (22.6)	2 (6.9)	13 (15.7)	
AJCC stage (%)					0.531
III	6 (26.1)	12 (38.7)	8 (27.6)	26 (31.3)	
IV	17 (73.9)	19 (61.3)	21 (72.4)	57 (68.7)	
T stage (%)					0.949
T1	2 (8.7)	5 (16.1)	5 (17.2)	12 (14.5)	
T2	10 (43.5)	11 (35.5)	9 (31.0)	30 (36.1)	
T3	5 (21.7)	7 (22.6)	6 (20.7)	18 (21.7)	
T4a	5 (21.7)	7 (22.6)	9 (31.0)	21 (25.3)	
T4b	1 (4.3)	1 (3.2)	0 (0)	2 (2.4)	
N stage (%)					0.895
N0	1 (4.3)	3 (9.7)	5 (17.2)	9 (10.8)	
N1	7 (30.4)	9 (29.0)	6 (20.7)	22 (26.5)	
N2a	2 (8.7)	1 (3.2)	1 (3.4)	4 (4.8)	
N2b	8 (34.8)	10 (32.3)	11 (37.9)	29 (34.9)	
N2c	5 (21.7)	8 (25.8)	6 (20.7)	19 (22.9)	

AJCC, American Joint Committee on Cancer. AJCC stage, Pretreatment clinical AJCC stage; T stage, Pretreatment clinical T stage; N stage, Pretreatment clinical N stage.

### Radiographic and pathological evaluation

**Figure 2:**
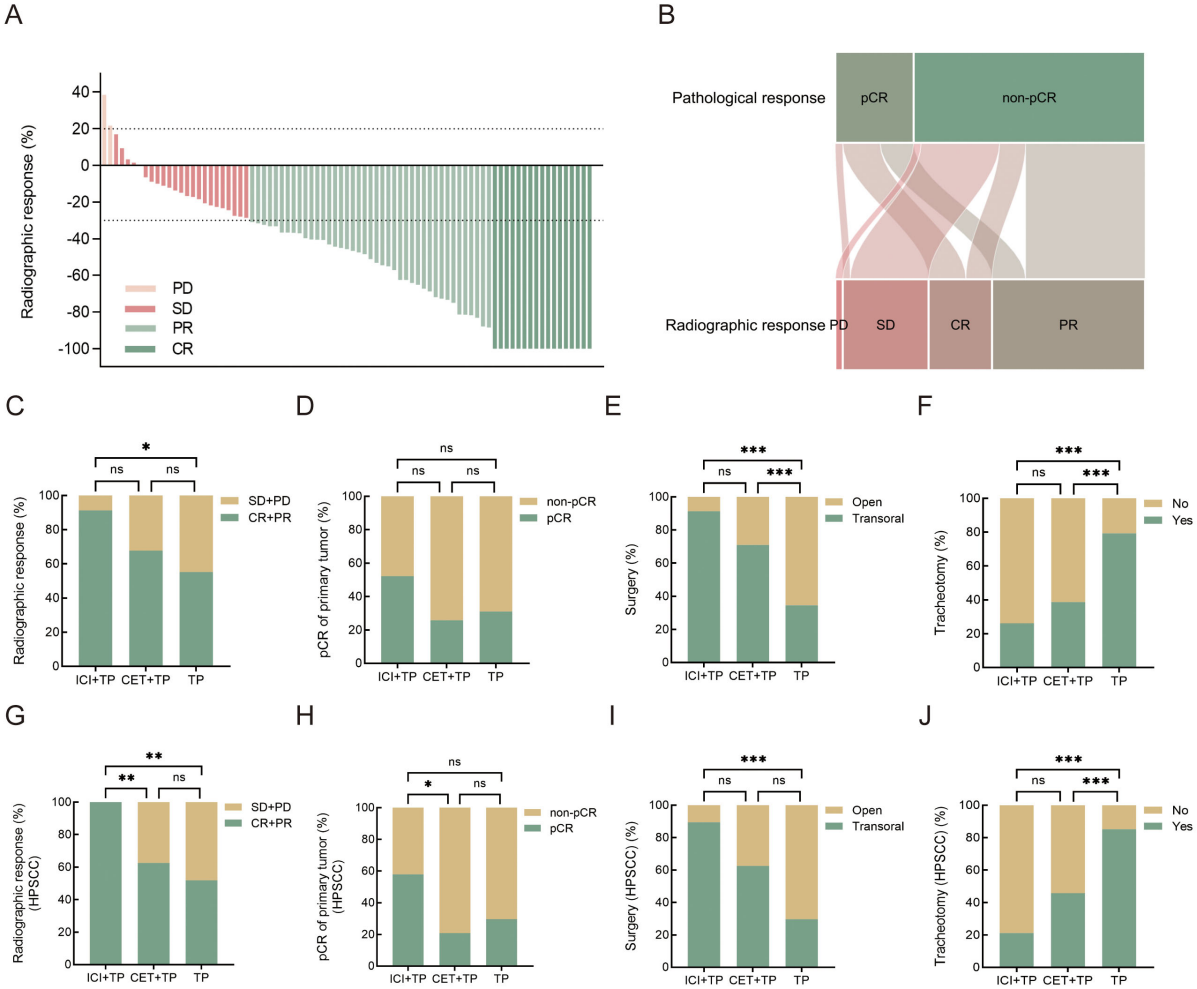
**(A)** Waterfall plot depicting best radiographic response per RECIST 1.1 criteria (n=83). Each vertical bar represents an individual patient. **(B)** Sankey plot illustrating the relationship between radiographic response and pathologic response. Comparative analysis of treatment groups (ICI+TP, CET+TP, and TP) in locally advanced OPHSCC **(C-F)** and HPSCC **(G-J)** patients assessed radiographic response, pathologic response of the primary tumor, surgical intervention rates, and tracheostomy rates. *p<0.05, **p<0.01, ***p<0.001, ns: no significance. Statistical analyses were performed by Chi-square test or Fisher’s exact test. CR, complete response; PR, partial response; SD, stable disease; PD, progressive disease; pCR, complete pathological response; ICI+TP, pembrolizumab combined with paclitaxel and cisplatin; CET+TP, cetuximab combined with paclitaxel and cisplatin; TP, paclitaxel and cisplatin; OPHSCC, oropharyngeal and hypopharyngeal squamous cell carcinoma; HPSCC, hypopharyngeal squamous cell carcinoma.

Radiographic assessment revealed an ORR of 91.3% (21/23) in the ICI+TP group, including 30.4% (7/23) CR and 60.9% (14/23) PR. The CET+TP group achieved an ORR of 67.7% (21/31), with 19.4% (6/31) CR and 48.4% (15/31) PR, while the TP group showed an ORR of 55.2% (16/29), comprising 13.8% (4/29) CR and 41.4% (12/29) PR (**Table 2**) (**Figure 2A**). The ORR difference between ICI+TP and TP was statistically significant (**Figure 2C**). Pathological analysis demonstrated a pCR in both primary tumors and cervical lymph nodes for 25.3% (21/83) of patients, with 9.6% (8/83) achieving MPR and 65.1% (54/83) showing IPR. The ICI+TP group had a pCR rate of 30.4% (7/23) and an MPR rate of 17.4% (4/23), compared to 22.6% (7/31) pCR and 3.2% (1/31) MPR in the CET+TP group, and 24.1% (7/29) pCR and 10.3% (3/29) MPR in the TP group. No significant intergroup differences in pCR rates were observed (**Table 2**). The relationship between pathologic response and radiographic response was showed in **Figure 2B**. Multivariate analyses identified pretreatment lymphocyte-to-monocyte ratio (pre-LMR) (OR = 1.47, 95% CI: 1.09-1.99, *p* = 0.012) as an independent predictor of pCR in OPHSCC patients receiving neoadjuvant therapy (**Supplementary Table S1**). Primary tumor pCR occurred in 34.9% (29/83) of all patients, with rates of 52.2% (12/23) in the ICI+TP, 25.8% (8/31) in the CET+TP, and 31% (9/29) in the TP, showing no significant intergroup differences (**Table 2**) (**Figure 2D**).

**Table 2:** Treatment responses and surgical interventions in all patients.

Characteristic	ICI+TP (n=23)	CET+TP (n=31)	TP (n=29)	*P* value
Radiographic response (%)				0.13
CR	7 (30.4)	6 (19.4)	4 (13.8)	
PR	14 (60.9)	15 (48.4)	12 (41.4)	
SD	2 (8.7)	9 (29.0)	12 (41.4)	
PD	0 (0)	1 (3.2)	1 (3.4)	
ORR (%)	21 (91.3)	21 (67.7)	16 (55.2)	0.016
Pathological response (%)				0.813
pCR	7 (30.4)	7 (22.6)	7 (24.1)	
non-pCR	16 (69.6)	24 (77.4)	22 (75.9)	
MPR	4 (17.4)	1 (3.2)	3 (10.3)	
IPR	12 (52.2)	23 (74.2)	19 (65.5)	
Pathological response of primary tumor (%)				0.118
pCR	12 (52.2)	8 (25.8)	9 (31.0)	
non-pCR	11 (47.8)	23 (74.2)	20 (69.0)	
MPR	5 (21.7)	2 (6.5)	3 (10.3)	
IPR	6 (26.1)	21 (67.7)	17 (58.6)	
Pathological response of lymph nodes (%)				0.817
pCR	11 (47.8)	12 (38.7)	12 (41.4)	
non-pCR	12 (52.2)	19 (61.3)	17 (58.6)	
MPR	2 (8.7)	2 (6.5)	1 (3.4)	
IPR	10 (43.5)	17 (54.8)	16 (55.2)	
Surgery (%)				0.000
Transoral	21 (91.3)	22 (71.0)	10 (34.5)	
Open	2 (8.7)	9 (29.0)	19 (65.5)	
Tracheotomy (%)				0.000
Yes	6 (26.1)	12 (38.7)	23 (79.3)	
No	17(73.9)	19 (61.3)	6 (20.7)	

CR, complete response, PR, partial response; SD, stable disease; PD, progressive disease; ORR, objective response rate; pCR, complete pathological response; MPR, major pathological response; IPR, incomplete pathological response.

Bold values indicate statistical significance.

**Figure 3:**
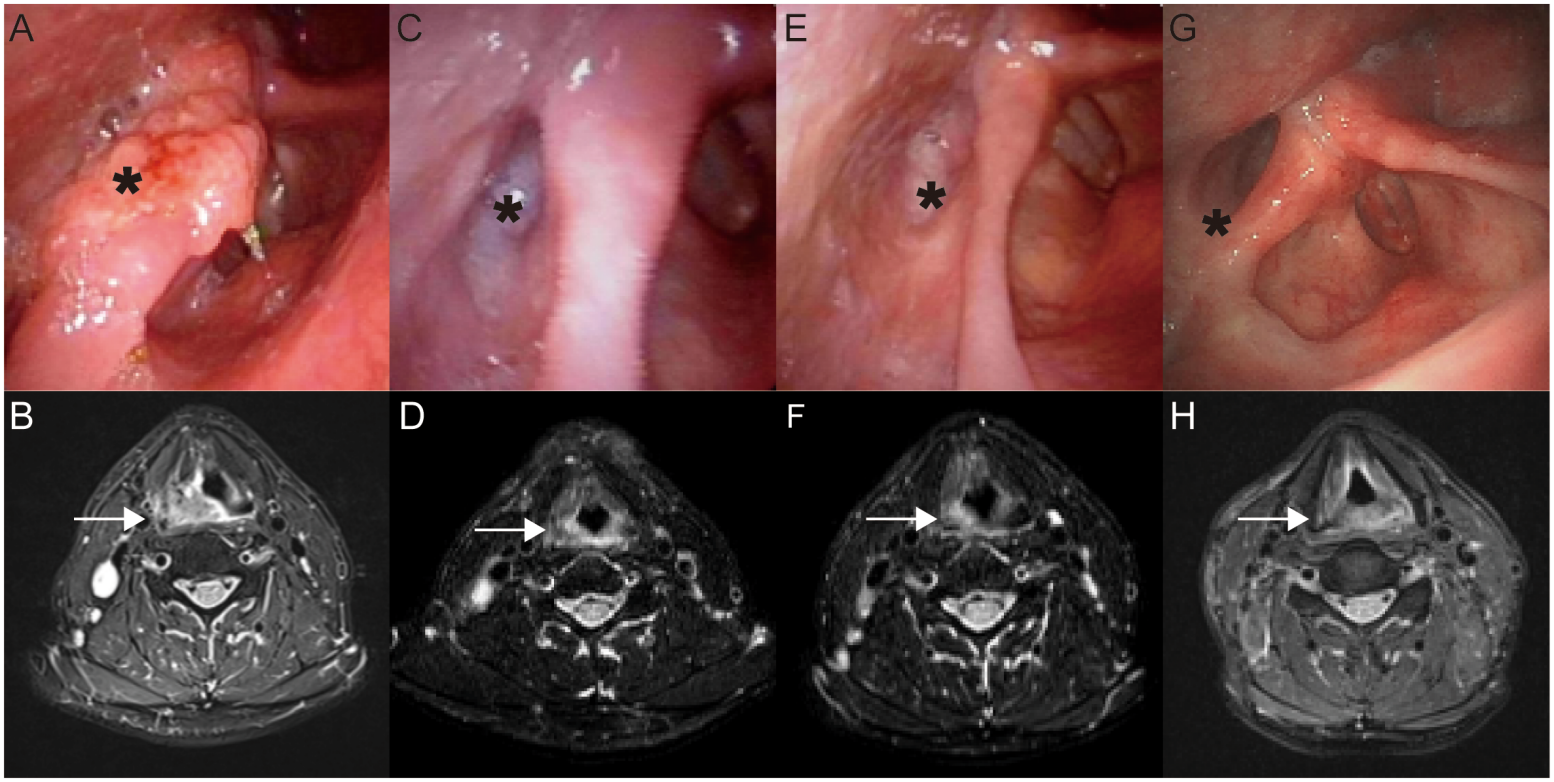
A representative case of neoadjuvant chemoimmunotherapy. A 56-year-old male patient was diagnosed with hypopharyngeal squamous cell carcinoma (T2N2bM0, stage IVA, AJCC staging). Pre-treatment laryngoscopy **(A)** revealed a cauliflower-like neoplasm at the lateral margin of the right arytenoid and right pyriform sinus (indicated by a black asterisk). MRI of the larynx **(B)** demonstrated a mass (22 mm × 16 mm) in the right pyriform sinus-supraglottic region (indicated by a white arrow), accompanied by multiple enlarged lymph nodes (42 mm × 16 mm) on the right side. The patient was subjected to neoadjuvant therapy with pembrolizumab (200 mg, day 1), paclitaxel (135 mg/m², day 2), and cisplatin (35 mg/m², days 2–4). Follow-up laryngoscopy **(C)** after the first cycle of neoadjuvant therapy showed mucosal hyperemia with minimal effusion in the right pyriform sinus (indicated by a black asterisk). MRI **(D)** revealed a reduction in the primary lesion to approximately 14 mm × 8 mm (indicated by a white arrow), with lymph node size decreasing to 14 mm × 11 mm. After the second cycle, laryngoscopy **(E)** demonstrated mucosal roughness in the right pyriform sinus (indicated by a black asterisk), while MRI **(F)** showed further regression of the primary lesion to 12 mm × 8 mm (indicated by a white arrow), with the largest lymph node diameter reduced to 7 mm. Imaging assessment indicated a PR. The patient subsequently underwent transoral resection of the right pyriform sinus lesion and right cervical lymph node dissection. Postoperative pathology revealed lymphocyte and plasma cell infiltration in the primary site with no residual tumor, while lymph nodes exhibited chemotherapy-induced changes without evidence of malignancy, achieving a pCR. Adjuvant radiotherapy was administered postoperatively. During regular outpatient follow-up, laryngoscopy **(G)** and MRI **(H)** at 19 months post-surgery showed no evidence of tumor recurrence or residual disease.

Among patients with HPSCC, the ICI+TP regimen achieved a 100% ORR, surpassing both the CET+TP and TP groups (**Figure 2G**). A representative case of neoadjuvant chemoimmunotherapy is illustrated in **Figure 3**. The pCR rates for primary tumors reached 57.9% (11/19) with ICI+TP, compared to 20.8% (5/24) for CET+TP and 29.6% (8/27) for TP alone, with a statistically significant difference between ICI+TP and CET+TP (**Supplementary Table S2**) (**Figure 2H**).

### Surgical treatments

After neoadjuvant therapy, transoral surgery (TOS) was performed in 91.3% of ICI+TP cases, 71% of CET+TP cases, and 34.5% of TP cases, with significant differences between ICI+TP vs. TP and CET+TP vs. TP (*p*<0.05). Tracheostomy rates were lower in the ICI+TP group (26.1%) than in the CET+TP (38.7%) and TP (79.3%) groups, showing significant differences for ICI+TP vs. TP and CET+TP vs. TP (*p*<0.05) (**Table 2**) (**Figures 2E, F**). In HPSCC patients, TOS rates reached 89.5% in the ICI+TP group, with a tracheostomy rate of 21.1%, demonstrating a significant difference compared to the TP group (*p*<0.05) but not the CET+TP group (*p*>0.05) (**Figures 2I, J**). Laryngeal preservation rates were 100% in the ICI+TP group, 87.5% in the CET+TP group, and 77.8% in the TP group, with no significant intergroup differences (*p*>0.05) (**Supplementary Table S2**).

### Survival analysis

**Figure 4:**
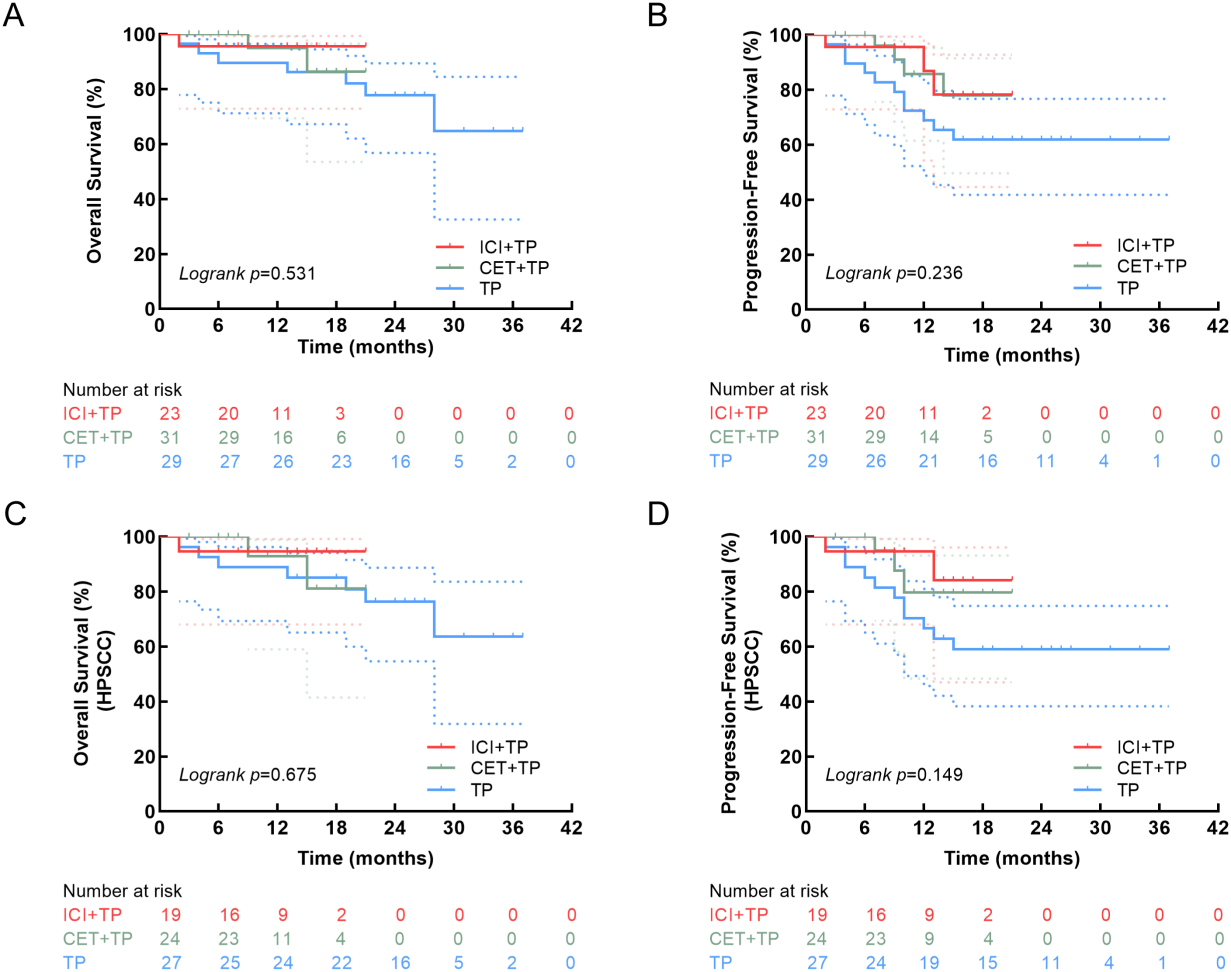
Kaplan-Meier curves comparing the overall survival **(A)** and progression-free survival **(B)** of the ICI+TP, CET+TP, and TP groups in the entire cohort. Corresponding analyses for overall survival **(C)** and progression-free survival **(D)** appear for the HPSCC patient subgroup. HPSCC: hypopharyngeal squamous cell carcinoma.

Median follow-up was 10 months (IQR, 6–17 months) for ICI+TP, 13 months (IQR, 7–16 months) for CET+TP, and 24 months (IQR, 18–27 months) for TP. The 1-year OS rates were 95.7%, 95.0%, and 89.7% for the ICI+TP, CET+TP, and TP, respectively. During follow-up, 10 deaths were recorded: one was due to postoperative asphyxia, one was attributed to COVID-19, and eight were caused by tumor recurrence or distant metastasis. One-year PFS rates were 87.0% (ICI+TP), 85.7% (CET+TP), and 69.0% (TP). No statistically significant differences in PFS or OS were observed within current follow-up duration among the three groups, as illustrated in **Figure 4**. Multivariate analysis identified open surgery (HR = 4.69, 95% CI: 1.42-15.45, *p* = 0.011) and larger pretreatment tumor diameter (HR = 1.76, 95% CI: 1.29-2.40, *p*<0.001) as independent predictors of shorter PFS. The analysis also showed that increased pretreatment tumor diameter (HR = 1.51, 95%CI: 1.10-2.08, p=0.011) independently predicted worse OS (**Table 3**).

**Table 3:** Univariate and multivariate COX regression models for predicting progression-free survival and overall survival.

Variable	Progression-free survival				Overall survival			
	Univariate analysis		Multivariate analysis		Univariate analysis		Multivariate analysis	
	HR (95% CI)	*p*	HR (95% CI)	*p*	HR (95% CI)	*p*	HR (95% CI)	*p*
Age, years								
<65	1 (Reference)				1 (Reference)			
≥65	1.95 (0.77-4.92)	0.158			2.86(0.81-10.14)	0.104		
Gender								
Male	1 (Reference)				1 (Reference)			
Female	0.39 (0.05-2.97)	0.366			0.04 (0.00-349.66)	0.490		
Primary site								
Oropharynx	1 (Reference)				0.04 (0.00-113.89)	0.425		
Hypopharynx	1.63 (0.38-7.11)	0.514			1 (Reference)			
AJCC stage								
III	1 (Reference)				1 (Reference)			
IV	2.28 (0.66-7.86)	0.194			1.55 (0.33-7.37)	0.579		
T stage								
T1-2	1 (Reference)		1 (Reference)		1 (Reference)			
T3-4	2.83 (1.01-7.94)	**0.048**	0.42 (0.10-1.78)	0.239	1.55 (0.43-5.56)	0.499		
N stage								
N0-1	1 (Reference)				1 (Reference)			
N2-3	1.09 (0.41-2.89)	0.871			1.32 (0.34-5.15)	0.689		
Therapy regimen								
ICI+TP	1 (Reference)				1 (Reference)			
CET+TP	0.96 (0.22-4.30)	0.959			1.50 (0.14-16.55)	0.741		
TP	2.17 (0.60-7.82)	0.235			2.83 (0.33-24.34)	0.344		
Surgery								
transoral	1 (Reference)		1 (Reference)		1 (Reference)			
open	3.92 (1.40-11.03)	**0.009**	4.69 (1.42-15.45)	**0.011**	4.45 (0.92-21.57)	0.064		
Radiographic response								
SD+PD	1 (Reference)				1 (Reference)			
CR+PR	0.77 (0.30-1.99)	0.595			0.65 (0.18-2.29)	0.498		
Pathological response								
non-pCR	1 (Reference)				1 (Reference)			
pCR	0.36 (0.08-1.58)	0.177			1.07 (0.22-5.22)	0.934		
Tracheotomy								
Yes	1 (Reference)				1 (Reference)			
no	0.34 (0.11-1.04)	0.060			0.42 (0.085-2.09)	0.292		
Radiotherapy								
Yes	1 (Reference)				1 (Reference)			
no	1.33 (0.30-5.84)	0.707			1.00 (0.12-8.08)	0.998		
pre-LMR	0.72 (0.51-1.02)	0.067			0.55 (0.30-1.01)	0.052		
pre-PNI	0.93 (0.85-1.02)	0.140			0.84 (0.74-0.96)	**0.008**	0.86 (0.76-0.99)	0.033
Pretreatment tumor diameter	1.56 (1.25-1.94)	**<0.001**	1.76 (1.29-2.40)	**<0.001**	1.63 (1.20-2.20)	**0.002**	1.51 (1.10-2.08)	**0.011**

ICI+TP, pembrolizumab combined with paclitaxel and cisplatin; CET+TP, cetuximab combined with paclitaxel and cisplatin; TP, paclitaxel and cisplatin; CR, complete response, PR, partial response; SD, stable disease; PD, progressive disease; pCR, complete pathological response; pre-LMR, pretreatment lymphocyte-to-monocyte ratio; pre-PNI, pretreatment prognostic nutritional index.

Variables meeting *p* < 0.05 were incorporated into the multivariate model.

The C-index for overall survival (OS) was 0.81 (95% confidence interval: 0.72-0.90), and the C-index for progression-free survival (PFS) was 0.79 (95% confidence interval: 0.74-0.85). The overall models for both OS and PFS, as well as all individual covariates, satisfied the proportional hazards (PH) assumption.

The Bonferroni method was applied to correct for multiple testing of the variables included in the model. In the multivariate COX regression analyses for predicting PFS, the corrected significance level (α) was established at 0.05/3≈0.017. For the multivariate COX regression models evaluating OS, the corrected significance level (α) was defined as 0.05/2 = 0.025.

Bold values indicate statistical significance.

### Treatment-related adverse events

Treatment-related adverse events were observed in 75 patients. The most frequently reported events were anemia (55/75, 73.3%), followed by hypokalemia (29/75, 38.7%), fatigue (28/75, 37.3%), granulocytopenia (23/75, 30.7%), nausea (22/75, 29.3%), and vomiting (10/75, 13.3%). Comprehensive data on adverse events for each treatment group and immune-related adverse events appear in **Supplementary Tables S3**, **S4**. Most adverse events were grade 1–2 in severity. Among patients receiving immune checkpoint inhibitors, two grade 3 events were observed: one case of immune-related hypothyroidism and one of hypokalemia. Both patients underwent specialist consultations, and the hypothyroidism was managed with levothyroxine replacement therapy, while hypokalemia resolved with potassium supplementation.

## Discussion

Locally advanced OPHSCC remains clinically challenging to manage, with treatment goals focused on enhancing both therapeutic outcomes and patient quality of life. Recent investigations into neoadjuvant PD-1 inhibitors have broadened treatment options for this aggressive malignancy. Reinvigorating pre-existing anti-tumor immunity and initiating novel tumor-specific immune responses are the fundamental basis for the efficacy of PD-1 inhibitors. PD-1 inhibitors bind specifically to PD-1 molecules on T cell surfaces via their antigen-binding fragments. By doing so, they occupy the ligand-binding pocket of PD-1, preventing PD-L1/PD-L2 (expressed on tumor or immune cells) from interacting with PD-1 and effectively blocking inhibitory signal transmission. Following PD-1 inhibitor treatment, newly expanded T cell clones recruit tumor-infiltrating lymphocytes (TILs) into the TME. These TILs initiate targeted immune responses by specifically recognizing tumor antigens. They also secrete large amounts of cytokines (e.g., IFN-γ and TNF-α) and release cytotoxic granules to eliminate tumor cells. Additionally, T cells recruit NK cells and macrophages into the TME, aiding in comprehensive tumor clearance. PD-1 inhibitors can also alter the differentiation trajectory of MDSCs, promoting their conversion into antigen-presenting cells. This enhances antigen presentation within the TME, thereby facilitating the initiation of effective anti-tumor immune responses (8, 19, 20). Against this mechanistic background, we evaluated neoadjuvant chemoimmunotherapy in resectable locally advanced OPHSCC. The regimen of preoperative pembrolizumab combined with paclitaxel and cisplatin achieved a high ORR and TOS rate. These results suggest its potential as an effective organ-preserving approach with favorable safety profiles.

Neoadjuvant chemoimmunotherapy effectively reduces tumor burden and achieves downstaging. In patients with early or locally advanced triple-negative breast cancer, adding camrelizumab to neoadjuvant chemotherapy significantly improved the pCR rate to 56.8% (21). Beyond breast cancer, neoadjuvant PD-1/PD-L1 inhibitors combined with chemotherapy have shown promising results in locally advanced non-small cell lung cancer. Specifically, they achieved a pCR rate of 29%, a MPR rate of 42.2%, and a surgical resection rate of 75% (22). For resectable locally advanced HNSCC, clinical trials have evaluated neoadjuvant PD-1 inhibitors—either used as monotherapy or combined with paclitaxel/cisplatin or low-dose radiotherapy. These regimens achieved pCR rates of 37%-60.9%, MPR rates of 21.7%-74.1%, and ORR of 64.3%–96.7% (12, 23, 24). Additionally, Fang et al. (18) conducted a retrospective study of 156 patients with locally advanced laryngeal/hypopharyngeal squamous cell carcinoma who received neoadjuvant PD-1 inhibitors combined with nab-paclitaxel/cisplatin, reporting an ORR of 88.5% and a pCR rate of 23.1%. Our findings demonstrate that the ICI+TP achieved an ORR of 91.3% with 30.4% CR, superior to TP alone (ORR = 55.2%) and comparable to the CET+TP group (ORR = 67.7%). Among HPSCC patients, ICI+TP achieved a 100% ORR, surpassing both CET+TP (62.5%) and TP (51.9%). These findings support neoadjuvant chemoimmunotherapy as an effective treatment for locally advanced OPHSCC. The high ORR, including a substantial CR rate, suggests its capacity not only for disease control but possibly for complete tumor eradication in select cases, which may improve surgical outcomes. Pathological evaluation revealed comparable pCR rates across all three treatment regimens in the overall cohort. However, in locally advanced HPSCC, patients subjected to ICI+TP exhibited significantly higher primary-site pCR rates than those receiving CET+TP (57.9% vs. 20.8%). Further analysis revealed that patients with a high pre-LMR were more likely to achieve pCR. LMR is a recognized prognostic factor in diverse hematologic and solid malignancies. It serves as an indirect indicator of the host’s inflammatory state, aligning with the concept that inflammation contributes to cancer progression. Notably, in the HPV-positive HNSCC cohort, a high LMR was associated with better clinical outcomes (25). Derived from routine blood tests, LMR can be monitored repeatedly before, during, and after treatment, providing real-time insight into changes in a patient's condition. However, this metric is susceptible to interference and cannot directly capture the local characteristics of the TME. Phenotypic and functional changes in circulating T-cell subsets may reflect anti-tumor immune activity more precisely than LMR. Studies indicate that PD-1^+^KLRG1^-^CD8^+^ T cells present in peripheral blood at baseline or shortly after PD-1 inhibitor therapy correlate significantly with pathological response, suggesting their potential as predictive biomarkers (26). Additionally, serum cytokine levels, as signaling molecules between immune cells, reflect the inflammatory status and immune balance within the TME. For example, TGFβ plays a key role in activating regulatory T cells (Treg) and suppressing NK cells. STAT3 activation, mediated by IL-6, inhibits the activation of T lymphocytes, tumor-associated macrophages (TAMs), NK cells, and neutrophils. It also impairs dendritic cell maturation, and is strongly associated with poor survival and increased recurrence in HNSCC patients. The IL-10 immunosuppressive pathway operates via the transcription factor STAT3, leading to reduced IL-12 expression and enhanced Treg differentiation in the TME (19). The association between pre-LMR and pCR identified in this study serves as an initial exploration. Future prospective studies should integrate more refined blood-based biomarkers, such as circulating T-cell subsets and cytokine profiles, to further elucidate the underlying mechanisms linking immune status to therapeutic efficacy. Moreover, in locally advanced OPHSCC patients receiving neoadjuvant chemoimmunotherapy, primary lesions exhibited a higher pCR rate (52.2%) than metastatic lymph nodes (47.8%), suggesting differential response patterns. Notably, residual tumor cells persisted in metastatic lymph nodes even when primary lesions achieved pCR. The underlying mechanism may involve the abundance of three cell populations in metastatic lymph nodes: terminally exhausted CD8^+^ T (T_Ex-term_) cells, PD-L1^+^ dendritic cells (DCs), and Treg cells with high expression of FOXP3 and exhaustion-associated genes. This immunosuppressive microenvironment inhibits the differentiation of progenitor exhausted T (T_PE_) cells into effector T cells, thereby limiting antitumor immune responses. Spatial multi-omics analysis revealed key characteristics of metastatic lymph nodes. T_PE_ and intermediate exhausted T (T_Ex-int_) cells are sparsely distributed here, lack effective cellular interactions, and show significantly lower PD-1 expression compared to their counterparts in regional lymph nodes. These findings further indicate a constrained immune activation state (26). Additional studies have shown dynamic changes in the cellular composition of metastatic lymph nodes, which harbor abundant macrophages. These macrophages exhibit an unfolded protein response and activate the protein kinase R-like endoplasmic reticulum kinase (PERK) signaling pathway. Through interactions with fibroblasts, they promote the formation of an extracellular matrix-enriched TME. This TME provides a protective niche for metastatic tumor cells, shielding them from immune therapy-induced attack (27). Therefore, for locally advanced OPHSCC patients, concurrent neck dissection should be performed during surgery—even after neoadjuvant therapy achieves radiological remission. Close monitoring is also needed to detect potential short-term systemic metastasis.

Neoadjuvant chemoimmunotherapy enhances TOS rates and preserves laryngeal function, enabling tracheostomy avoidance in select patients. In the pre-immunotherapy era, the standard treatment paradigm for resectable locally advanced OPHSCC involved primary radical surgery followed by adjuvant therapy based on pathological findings. Low-risk patients received adjuvant radiotherapy alone, while high-risk patients with positive margins or extracapsular nodal extension underwent concurrent chemoradiotherapy. Consequently, most patients with locally advanced HPSCC underwent open neck surgery, including partial laryngectomy, partial hypopharyngectomy, or total laryngectomy. For OPSCC patients, mandibulotomy and flap reconstruction were often required to achieve safe margins. Postoperatively, these patients inevitably faced impaired or lost vocal function, dysphagia, compromised craniofacial aesthetics, and permanent tracheostomies, significantly diminishing their quality of life. Extensive clinical data demonstrate that neoadjuvant treatment combining PD-1 inhibitors with paclitaxel and cisplatin exhibits favorable efficacy and tolerability for laryngeal preservation therapy in locally advanced HNSCC. A single-arm phase II clinical study reported an 88.9% laryngeal preservation rate with toripalimab plus paclitaxel and cisplatin in locally advanced laryngeal and hypopharyngeal cancers (2). Retrospective studies further show that neoadjuvant chemoimmunotherapy followed by surgery achieves postoperative laryngeal preservation rates of 92.9%-95% (28, 29), a TOS rate of 82.1% (29), and a 1-year laryngeal preservation rate of 96.8%. These rates are significantly higher than those of targeted therapy plus neoadjuvant chemotherapy (68.4%) and neoadjuvant chemotherapy alone (75%) (30). In this study, patients achieving CR or PR in the primary tumor underwent transoral endoscopic resection. This approach allowed precise pathological assessment of tumor response while preserving laryngeal function. After rigorous intraoperative hemostasis and edema evaluation, tracheostomy was avoided in select patients. Our data reveal a 91.3% TOS rate in locally advanced OPHSCC patients treated with ICI+TP, surpassing rates in the CET+TP (71.0%) and TP (34.5%) groups, with only 26.1% requiring tracheostomy. Subgroup analysis of locally advanced HPSCC revealed an 89.5% TOS rate and a 21.1% tracheostomy rate in the ICI+TP group. Notably, patients who underwent transoral endoscopic surgery had better postoperative functional recovery in respiration, swallowing, and phonation than open surgery patients, with significant quality-of-life benefits. A key driver for the enhanced feasibility of TOS is the effective induction of immunogenic tumor regression by PD-1 inhibitors. This is accompanied by significant tumor volume reduction, which is associated with a high ORR. Primary tumor shrinkage reduces invasion into adjacent critical anatomical structures. It transforms the tumor-normal tissue boundary from indistinct and infiltrative to relatively well-defined. This facilitates complete tumor resection during TOS while minimizing damage to normal structures, preserving pharyngeal integrity, and lowering the risk of postoperative swallowing dysfunction. Furthermore, Huang et al. (31) revealed that after PD-1/PD-L1 blockade therapy, tumor-specific memory CD8^+^ T cells derived from tumor-draining lymph nodes (TdLNs) differentiate and expand rapidly. These cells exhibit high tumor specificity and activity, enabling them to recognize and attack tumor cells. In turn, this contributes to sustained long-term antitumor immunity and reduces the risk of perioperative recurrence.

In the investigation of immunotherapy for locally advanced HNSCC, the primary objectives focus on reduce recurrence/metastasis risk and achieve survival benefits. However, multiple prior clinical trials have failed to demonstrate significant survival improvements. The KEYNOTE-412 randomized 804 patients with locally advanced HNSCC to receive concurrent chemoradiotherapy (CCRT) plus either pembrolizumab or placebo. Results showed no significant differences in median event-free survival (EFS) or OS between the two groups (32). Similarly, atezolizumab did not improve clinical outcomes for high-risk patients following multimodal curative therapy (33). In 2025, the KEYNOTE-689 trial marked a breakthrough as the first Phase III study to demonstrate EFS benefits and meet its primary endpoint in resectable locally advanced HNSCC. It confirmed that neoadjuvant pembrolizumab monotherapy (before surgery), followed by adjuvant and maintenance pembrolizumab (after postoperative radiotherapy or chemoradiotherapy), significantly prolonged median EFS. This benefit was observed in patients with a PD-L1 combined positive score (CPS) ≥10, CPS ≥1, and the overall population, while reducing the risk of disease progression, recurrence, or death (34, 35). These findings suggest that PD-1 inhibitors may play a specific role in targeting micrometastases or minimal residual disease following curative surgery. In our study, ICI+TP regimen did not significantly improve PFS or OS compared to CET+TP or TP. Thus, additional data accumulation and longer follow-up are needed to clarify the long-term survival impact of neoadjuvant chemoimmunotherapy in patients with locally advanced OPHSCC.

Several limitations of this study should be acknowledged. First, this was a single-center retrospective investigation, which may introduce inherent selection bias. Second, the small sample size limits the statistical power of our results, necessitating validation through larger prospective studies. Third, the short follow-up duration is another significant constraint, particularly for evaluating long-term treatment outcomes. The median follow-up time was too brief, and the number of events was too limited, making it impossible to calculate median OS or PFS. Thus, the survival outcomes reported here should therefore be considered preliminary, and potential long-term benefits require validation through extended follow-up. Finally, PD-L1 CPS testing and HPV infection data were not available in pembrolizumab-treated patients, which precluded further subgroup analyses stratified by CPS and HPV status. Despite these limitations, this study provides valuable preliminary evidence regarding the efficacy of neoadjuvant chemoimmunotherapy in locally advanced OPHSCC.

## Conclusion

In locally advanced OPHSCC, neoadjuvant pembrolizumab combined with paclitaxel and cisplatin showed a higher ORR and increased transoral surgery rates. Additionally, it preserved laryngeal function without increasing perioperative complications, demonstrating favorable efficacy and safety profiles. Given the present study’s limitations of small sample size and short follow-up duration, larger-scale prospective studies with extended follow-up are needed to comprehensively assess the benefits and risks of this treatment regimen, as well as to explore potential approaches for optimizing outcomes.

## Data Availability

The raw data supporting the conclusions of this article will be made available by the authors, without undue reservation.
