# Supplementary material for: Efficacy of neoadjuvant pembrolizumab combined with paclitaxel and cisplatin in locally advanced oropharyngeal and hypopharyngeal squamous cell carcinoma: a retrospective study
Source: Front Immunol. 2025 Nov 21;16:1690935. doi: 10.3389/fimmu.2025.1690935 (PMC12678248; doi:10.3389/fimmu.2025.1690935)
Supplement: Supplementary file 1 [file Table1.docx]

**Table S1** Univariate and multivariate logistic regression models for predicting pCR.

|  | **Univariate analysis** | |  | **Multivariate analysis** | |
| --- | --- | --- | --- | --- | --- |
|  | OR (95% CI) | *p* value |  | OR (95% CI) | *p value* |
| Age (years) |  |  |  |  |  |
| <65 | 1 (Reference) |  |  |  |  |
| ≥65 | 1.78 (0.65-4.85) | 0.263 |  |  |  |
| Gender |  |  |  |  |  |
| Male | 1 (Reference) |  |  |  |  |
| Female | 2.68 (0.65-11.12) | 0.174 |  |  |  |
| Primary site |  |  |  |  |  |
| Oropharynx | 1 (Reference) |  |  |  |  |
| Hypopharynx | 0.72 (0.19-2.64) | 0.622 |  |  |  |
| AJCC stage |  |  |  |  |  |
| Ⅲ | 1 (Reference) |  |  |  |  |
| Ⅳ | 0.88 (0.31-2.54) | 0.818 |  |  |  |
| T stage |  |  |  |  |  |
| T1-2 | 1 (Reference) |  |  |  |  |
| T3-4 | 0.70 (0.26-1.91) | 0.489 |  |  |  |
| N stage |  |  |  |  |  |
| N0-1 | 1 (Reference) |  |  |  |  |
| N2-3 | 0.96 (0.35-2.66) | 0.935 |  |  |  |
| Therapy regimen |  |  |  |  |  |
| ICI+TP | 1 (Reference) |  |  |  |  |
| CET+TP | 0.67 (0.19-2.27) | 0.516 |  |  |  |
| TP | 0.73 (0.21-2.49) | 0.612 |  |  |  |
| Radiographic response |  |  |  |  |  |
| SD+PD | 1 (Reference) |  |  | 1 (Reference) |  |
| CR+PR | 5.60 (1.19-26.28) | **0.029** |  | 5.65 (1.15-27.75) | 0.033 |
| pre-LMR | 1.46 (1.10-1.93) | **0.008** |  | 1.47 (1.09-1.99) | **0.012** |
| pre-PNI | 0.921 (0.83-1.02) | 0.110 |  |  |  |

pCR: complete pathological response; AJCC stage: Pretreatment clinical AJCC stage; T stage: Pretreatment clinical T stage; N stage: Pretreatment clinical N stage; ICI+TP: pembrolizumab combined with paclitaxel and cisplatin; CET+TP: cetuximab combined with paclitaxel and cisplatin; TP: paclitaxel and cisplatin; pre-LMR: pretreatment lymphocyte-to-monocyte ratio; pre-PNI: pretreatment prognostic nutritional index; CR: complete response, PR: partial response; SD: stable disease; PD: progressive disease.

The Bonferroni method was applied to correct for multiple testing of the variables included in the model. The corrected significance level (α) was set to 0.05/2=0.025.

**Table S2** Treatment responses and surgical interventions in HPSCC patients.

|  | ICI+TP (%) (n=19) | CET+TP (%) (n=24) | TP (%)  (n=27) | *p* value |
| --- | --- | --- | --- | --- |
| ORR (%) | 19 (100) | 15 (62.5) | 14 (51.9) | **0.001** |
| Pathological response (%) |  |  |  | 0.318 |
| pCR | 7 (36.8) | 4 (16.7) | 6 (22.2) |  |
| non-pCR | 12 (63.2) | 20 (83.3) | 21 (77.8) |  |
| MPR | 3 (15.8) | 1 (4.2) | 2 (7.4) |  |
| IPR | 9 (47.4) | 19 (79.2) | 19 (70.4) |  |
| Pathological response of primary tumor (%) |  |  |  | **0.032** |
| pCR | 11 (57.9) | 5 (20.8) | 8 (29.6) |  |
| non-pCR | 8 (42.1) | 19 (79.2) | 19 (70.4) |  |
| MPR | 4 (21.1) | 1 (4.2) | 2 (7.4) |  |
| IPR | 4 (21.1) | 18 (75.0) | 17 (63.0) |  |
| Pathological response of lymph nodes (%) |  |  |  | 0.509 |
| pCR | 10 (52.6) | 9 (37.5) | 10 (37.0) |  |
| non-pCR | 9 (47.4) | 15 (62.5) | 17 (63.0) |  |
| MPR | 1 (5.3) | 2 (8.3) | 1 (3.7) |  |
| IPR | 8 (42.1) | 13 (54.2) | 16 (59.3) |  |
| Surgery (%) |  |  |  | **0.000** |
| Transoral | 17 (89.5) | 15 (62.5) | 8 (29.6) |  |
| Open | 2 (10.5) | 9 (37.5) | 19 (70.4) |  |
| Larynx preservation rate (%) | 19 (100) | 21 (87.5) | 21 (77.8) | 0.064 |
| Tracheotomy (%) |  |  |  | **0.000** |
| Yes | 4 (21.1) | 11 (45.8) | 23 (85.2) |  |
| No | 15 (78.9) | 13 (54.2) | 4 (14.8) |  |

HPSCC: hypopharyngeal squamous cell carcinoma; ICI+TP: pembrolizumab combined with paclitaxel and cisplatin; CET+TP: cetuximab combined with paclitaxel and cisplatin; TP: paclitaxel and cisplatin; ORR: objective response rate; pCR: complete pathological response; MPR: major pathological response; IPR: incomplete pathological response

**Table S3** Treatment-related adverse events

| Adverse events | ICI+TP (n=23) | | CET+TP (n=31) | | TP (n=29) | | Total |
| --- | --- | --- | --- | --- | --- | --- | --- |
|  | Grade 1-2 | Grade  3-4 | Grade  1-2 | Grade  3-4 | Grade  1-2 | Grade  3-4 |  |
| Granulocytopenia | 5 | 0 | 9 | 3 | 4 | 2 | 23 |
| Anemia | 14 | 0 | 26 | 0 | 15 | 0 | 55 |
| Rash | 0 | 0 | 1 | 0 | 0 | 0 | 1 |
| Hypothyroidism | 2 | 1 | 2 | 0 | 1 | 0 | 6 |
| Hepatotoxicity | 0 | 0 | 0 | 0 | 0 | 0 | 0 |
| Nephrotoxicity | 3 | 0 | 1 | 0 | 1 | 0 | 5 |
| Hypokalemia | 6 | 1 | 11 | 1 | 9 | 1 | 29 |
| Fatigue | 5 | 0 | 17 | 0 | 6 | 0 | 28 |
| Nausea | 7 | 0 | 11 | 0 | 4 | 0 | 22 |
| Vomiting | 3 | 0 | 6 | 0 | 1 | 0 | 10 |

ICI+TP: pembrolizumab combined with paclitaxel and cisplatin; CET+TP: cetuximab combined with paclitaxel and cisplatin; TP: paclitaxel and cisplatin

**Table S4** Immune-related adverse events

|  | Any grade | Grade 1 | Grade 2 | Grade 3 | Grade 4 |
| --- | --- | --- | --- | --- | --- |
| Granulocytopenia | 5 | 2 | 3 | 0 | 0 |
| Anemia | 14 | 11 | 3 | 0 | 0 |
| Hypothyroidism | 3 | 1 | 1 | 1 | 0 |
| Nephrotoxicity | 3 | 1 | 2 | 0 | 0 |
| Hypokalemia | 7 | 5 | 1 | 1 | 0 |
| Fatigue | 5 | 5 | 0 | 0 | 0 |
| Nausea | 7 | 7 | 0 | 0 | 0 |
| Vomiting | 3 | 3 | 0 | 0 | 0 |

**Table S5** Subgroup evaluation by tumor site.

|  | ICI+TP (n=23) | |  | CET+TP (n=31) | |  | TP (n=29) | |  |
| --- | --- | --- | --- | --- | --- | --- | --- | --- | --- |
| Characteristic | Hypopharynx (n=19) | Oropharynx (n=4) | *p* value | Hypopharynx (n=24) | Oropharynx (n=7) | *p* value | Hypopharynx (n=27) | Oropharynx (n=2) | *p* value |
| Radiographic response (%) |  |  | **0.024** |  |  | 0.379 |  |  | 0.488 |
| CR+PR | 19 (100) | 2 (50) |  | 15 (62.5) | 6 (85.7) |  | 14 (51.9) | 2 (100) |  |
| SD+PD | 0 (0) | 2 (50) |  | 9 (37.5) | 1 (14.3) |  | 13 (48.1) | 0 (0) |  |
| Pathological response (%) |  |  | 0.273 |  |  | 0.302 |  |  | 0.431 |
| pCR | 7 (36.8) | 0 (0) |  | 4 (16.7) | 3 (42.9) |  | 6 (22.2) | 1 (50) |  |
| non-pCR | 12 (63.2) | 4 (100) |  | 20 (83.3) | 4 (57.1) |  | 21 (77.8) | 1 (50) |  |
| Pathological response of primary tumor (%) |  |  | 0.317 |  |  | 0.335 |  |  | 0.532 |
| pCR | 11 (57.9) | 1 (25) |  | 5 (20.8) | 3 (42.9) |  | 8 (29.6) | 1 (50) |  |
| non-pCR | 8 (42.1) | 3 (75) |  | 19 (79.2) | 4 (57.1) |  | 19 (70.4) | 1 (50) |  |
| Pathological response of lymph nodes (%) |  |  | 0.59 |  |  | 1 |  |  | 0.163 |
| pCR | 10 (52.6) | 1 (25) |  | 9 (37.5) | 3 (42.9) |  | 10 (37) | 2 (100) |  |
| non-pCR | 9 (47.4) | 3 (75) |  | 15 (62.5) | 4 (57.1) |  | 17 (63) | 0 (0) |  |
| Surgery (%) |  |  | 1 |  |  | 0.077 |  |  | 0.111 |
| Transoral | 17 (89.5) | 4 (100) |  | 15 (62.5) | 7 (100) |  | 8 (29.6) | 2 (100) |  |
| Open | 2 (10.5) | 0 (0) |  | 9 (37.5) | 0 (0) |  | 19 (70.4) | 0 (0) |  |
| Tracheotomy (%) |  |  | 0.27 |  |  | 0.201 |  |  | **0.037** |
| Yes | 4 (21.1) | 2 (50) |  | 11 (45.8) | 1 (14.3) |  | 23 (85.2) | 0 (0) |  |
| No | 15 (78.9) | 2 (50) |  | 13 (54.2) | 6 (85.7) |  | 4 (14.8) | 2 (100) |  |

**Table S6** Subgroup evaluation by AJCC stage.

|  | ICI+TP (n=23) | |  | CET+TP (n=31) | |  | TP (n=29) | |  |
| --- | --- | --- | --- | --- | --- | --- | --- | --- | --- |
| Characteristic | stage Ⅲ | stage Ⅳ | *p* value | stage Ⅲ | stage Ⅳ | *p* value | stage Ⅲ | stage Ⅳ | *p* value |
| Radiographic response (%) |  |  | 1 |  |  | 1 |  |  | 0.238 |
| CR+PR | 6 (100) | 15 (88.2) |  | 8 (66.7) | 13 (68.4) |  | 6 (75.0) | 10 (47.6) |  |
| SD+PD | 0 | 2 (11.8) |  | 4 (33.3) | 6 (31.6) |  | 2 (25.0) | 11 (52.4) |  |
| Pathological response (%) |  |  | 1 |  |  | 0.676 |  |  | 0.357 |
| pCR | 2 (33.3) | 5 (29.4) |  | 2 (16.7) | 5 (26.3) |  | 3 (37.5) | 4 (19.0) |  |
| non-pCR | 4 (66.7) | 12 (70.6) |  | 10 (83.3) | 14 (73.7) |  | 5 (62.5) | 17 (81.0) |  |
| Pathological response of primary tumor (%) |  |  | 1 |  |  | 1 |  |  | 0.209 |
| pCR | 3 (50.0) | 9 (52.9) |  | 3 (25.0) | 5 (26.3) |  | 4 (50.0) | 5 (23.8) |  |
| non-pCR | 3 (50.0) | 8 (47.1) |  | 9 (75.0) | 14 (73.7) |  | 4 (50.0) | 16 (76.2) |  |
| Pathological response of lymph nodes (%) |  |  | 1 |  |  | 1 |  |  | **0.003** |
| pCR | 3 (50.0) | 8 (47.1) |  | 5 (41.7) | 7 (36.8) |  | 7 (87.5) | 5 (23.8) |  |
| non-pCR | 3 (50.0) | 9 (52.9) |  | 7 (58.3) | 12 (63.2) |  | 1 (12.5) | 16 (76.2) |  |
| Surgery (%) |  |  | 1 |  |  | 0.418 |  |  | 0.083 |
| Transoral | 6 (100.0) | 15 (88.2) |  | 10 (83.3) | 12 (63.2) |  | 5 (62.5) | 5 (23.8) |  |
| Open | 0 (0) | 2 (11.8) |  | 2 (16.7) | 7 (36.8) |  | 3 (37.5) | 16 (76.2) |  |
| Tracheotomy (%) |  |  | 0.144 |  |  | 0.717 |  |  | 0.305 |
| Yes | 0 (0) | 6 (35.3) |  | 4 (33.3) | 8 (42.1) |  | 5 (62.5) | 18 (85.7) |  |
| No | 6 (100.0) | 11 (64.7) |  | 8 (66.7) | 11 (57.9) |  | 3 (37.5) | 3 (14.3) |  |

**Table S7** Pairwise comparisons of Kaplan-Meier method

|  | Group | HR | CI | *p* value | Adjusted-*p* value |
| --- | --- | --- | --- | --- | --- |
| PFS | ICI+TP vs. CET+TP | 0.97 | 0.22-4.34 | 0.96 | 1.00 |
|  | ICI+TP vs.TP | 2.22 | 0.75-6.55 | 0.20 | 0.60 |
|  | CET+TP vs.TP | 2.26 | 0.82-6.25 | 0.14 | 0.43 |
|  |  |  |  |  |  |
| OS | ICI+TP vs. CET+TP | 1.45 | 0.15-14.32 | 0.76 | 1.00 |
|  | ICI+TP vs.TP | 2.84 | 0.62-13.11 | 0.27 | 0.81 |
|  | CET+TP vs.TP | 1.66 | 0.41-6.71 | 0.48 | 1.00 |
|  |  |  |  |  |  |
| PFS-HPSCC | ICI+TP vs. CET+TP | 1.31 | 0.23-7.61 | 0.76 | 1.00 |
|  | ICI+TP vs.TP | 3.14 | 1.01-9.72 | 0.11 | 0.33 |
|  | CET+TP vs.TP | 2.42 | 0.83-7.05 | 0.15 | 0.46 |
|  |  |  |  |  |  |
| OS-HPSCC | ICI+TP vs. CET+TP | 1.68 | 0.17-16.30 | 0.67 | 1.00 |
|  | ICI+TP vs.TP | 2.47 | 0.51-11.98 | 0.34 | 1.00 |
|  | CET+TP vs.TP | 1.36 | 0.32-5.81 | 0.68 | 1.00 |

Pairwise comparisons were performed using the log-rank test, with Bonferroni correction applied to adjust the p-values.
